# High-Speed Nanomechanical Mapping of the Early Stages of Collagen Growth by Bimodal Force Microscopy

**DOI:** 10.1021/acsnano.0c10159

**Published:** 2021-01-07

**Authors:** Victor G. Gisbert, Simone Benaglia, Manuel R. Uhlig, Roger Proksch, Ricardo Garcia

**Affiliations:** † Instituto de Ciencia de Materiales de Madrid, CSIC, c/Sor Juana Inés de la Cruz 3, 28049 Madrid, Spain; ‡ Asylum Research an Oxford Instruments Company, Santa Barbara, California 93117, United States

**Keywords:** high-speed AFM, bimodal AFM, nanomechanics, viscoelasticity, collagen

## Abstract

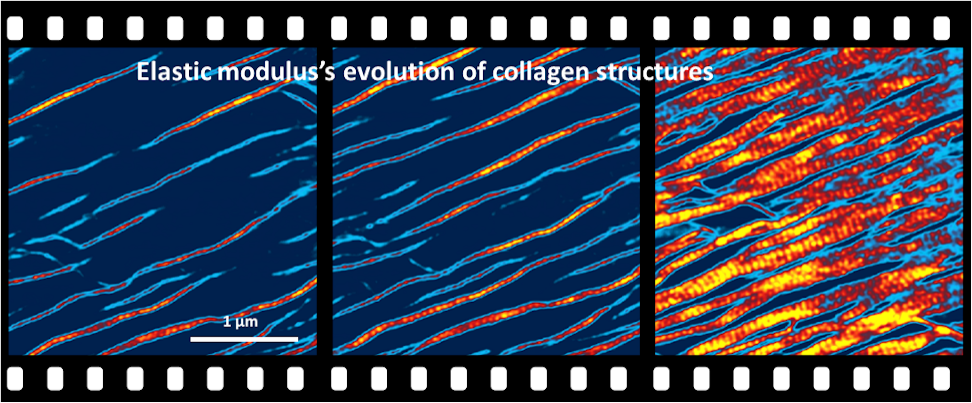

High-speed atomic force microscopy (AFM) enabled the imaging of protein interactions with millisecond time resolutions (10 fps). However, the acquisition of nanomechanical maps of proteins is about 100 times slower. Here, we developed a high-speed bimodal AFM that provided high-spatial resolution maps of the elastic modulus, the loss tangent, and the topography at imaging rates of 5 fps. The microscope was applied to identify the initial stages of the self-assembly of the collagen structures. By following the changes in the physical properties, we identified four stages, nucleation and growth of collagen precursors, formation of tropocollagen molecules, assembly of tropocollagens into microfibrils, and alignment of microfibrils to generate microribbons. Some emerging collagen structures never matured, and after an existence of several seconds, they disappeared into the solution. The elastic modulus of a microfibril (∼4 MPa) implied very small stiffness (∼3 × 10^–6^ N/m). Those values amplified the amplitude of the collagen thermal fluctuations on the mica plane, which facilitated microribbon build-up.

The relationship between the mechanical properties of proteins and their function is still emerging.^1−3^ The dynamics of protein on surfaces and their self-assembly has an impact in several biological processes, ranging from the polymerization of the actin cytoskeleton in cells to the amyloid fibrils involved in some neurodegenerative diseases. Until the development of high-speed atomic force microscopy (HS-AFM), it was not possible to follow in real time and with high-spatial resolution the evolution of those processes.^4−16^ HS-AFM images have a key limitation, and the data do not provide information about the mechanical properties of proteins such as the elastic modulus or the loss tangent. High-resolution maps of mechanical properties are obtained using AFM methods that operate at imaging rates about 100 times slower.^17^ To overcome this limitation, we combined the nanomechanical mapping features of bimodal AFM^18−20^ with the time-resolution capabilities of HS-AFM.^4−6^ The HS bimodal AFM characterized the early stages of the self-assembly of collagen fibrils on a mica surface by providing time-resolved and high-spatial resolution maps of the topography and the mechanical properties of collagen structures during growth.

Collagen is the most abundant structural protein in humans and vertebrates.^21−23^ The assembly of collagen fibrils plays relevant roles in a variety of biological processes.^22,23^ Several AFM (topography) studies on the formation of collagen fibrils and microribbons of different types of collagen were reported.^24−28^ Those studies were followed by AFM-based force spectroscopy experiments aimed to measure the Young’s modulus of wet collagen microribbons.^29−32^ The elastic properties of collagen fibrils were also determined from persistence length values deduced from AFM height images.^33^ Those studies lacked the temporal and mechanical property resolutions required to identify the early stages of collagen self-assembly and microfibril formation.

HS bimodal AFM shares some concepts, theory, and experimental setup with low-speed bimodal^18,34−39^ AFM. However, the HS bimodal configuration departs from recent trends based on the simultaneous implementation of two feedback loops.^19,20,37^ We implemented a bimodal AFM open-loop configuration that simplified the feedback loops without penalizing the quantitative accuracy in the determination of mechanical properties.

## Results and Discussion

### Theory of HS Bimodal AFM

The theory for HS bimodal AFM follows the conventional bimodal AFM theory by applying energy balance and virial principles.^40^ For a tip ended in a half-sphere with the radius *R*, the elastic modulus (Young’s modulus) *E*, and the loss tangent tan ρ were determined by
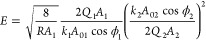
1
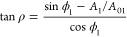
2The free amplitudes *A*_0*i*_ were established before the measurements, and *A*_2_ and the phase shifts ϕ_*i*_ were determined at the same time as the topography (see Materials and Methods for the definitions of the other parameters). Thus, bimodal AFM mapping did not introduce any time delay with respect to HS-AFM imaging. The above features are the key points for achieving quantitative mechanical property mapping at high-speed and high-spatial resolution. Other relevant technical features are akin to HS-AFM operation such as the use of small cantilevers and a high-frequency *z*-motion.^5^

The accuracy of the eq 1 was validated by numerical simulations provided by an open-source code.^41^ In the present bimodal AFM configuration, the force exerted by the tip is perpendicular to the sample. Therefore, the component of the elastic modulus tensor reported here is the one perpendicular to the sample surface. Equation 2 describes the loss tangent associated with the first mode. This expression did not take into account the mechanical energy transferred from this mode to the second mode or higher harmonics. As a consequence, eq 2 should not be applied to compare loss tangent values obtained on stiff (∼50 GPa, mica) and soft (∼5 MPa, collagen) regions. However, eq 2 provides a good approximation to estimate the dissipative properties of the different collagen structures (see Supporting Information (SI)).

### High-Speed Nanomechanical Maps of Early-Stage Collagen Structures

**Figure 1:**
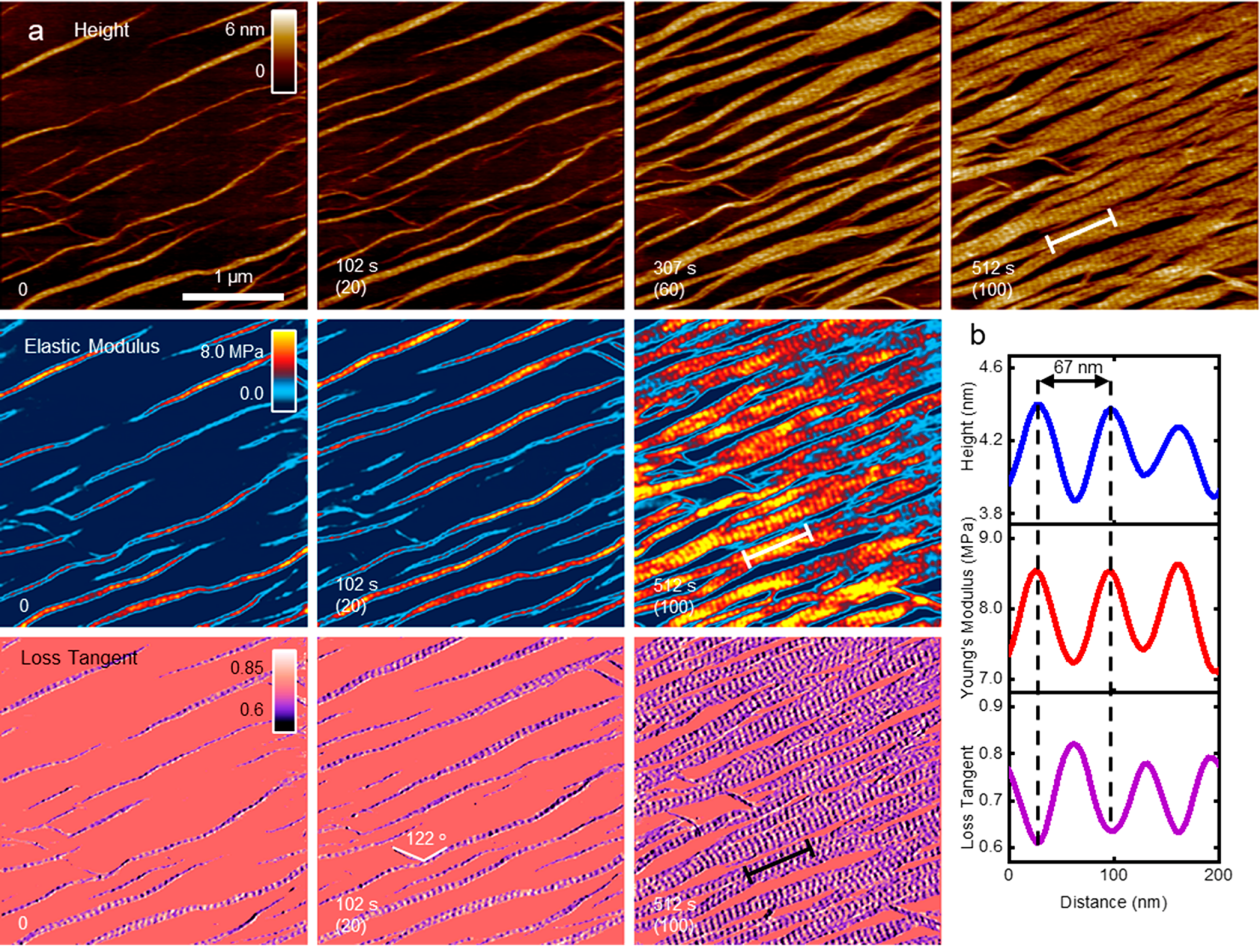
High-speed bimodal AFM maps of topography, elastic modulus, and loss tangent of collagen microribbons. (a) Sequence of topography, elastic modulus, and loss tangent maps showing collagen microribbon growth on mica (see Movie S1 in SI). The sequence captures the merging of and the alignment of the D-bands of several microribbons. The numbers in parentheses indicate the frame order in the video. (b) Cross sections along the lines marked in the frames recorded at *t* = 512 s. All of the properties oscillate with the periodicity of the D-band structure. Some of the cross section points were generated using a cubic interpolation regression obtained from 10 contiguous experimental pixels. The maps were obtained in buffer by applying a peak force on the microribbons of 1.2 nN (18 nN on mica). Imaging rate, 0.2 fps (512 × 512 pixels, scanline of 100 Hz). Additional bimodal AFM data: *f*_1_ = 132 kHz, *k*_1_ = 0.18 nN/nm, *Q*_1_ = 1.7; *f*_2_ = 1045 kHz, *k*_2_ = 12 nN/nm, *Q*_2_ = 5.3; *A*_01_ = 10 nm, *A*_02_ = 1.5 nm, and *A*_1_ = 9 nm.

First, we proceeded to identify the physical properties of the collagen structures that were previously imaged by electron microscopy and AFM such as microribbons.^23−28^Figure 1a shows a sequence of topography, elastic modulus, and loss tangent images lasting for 5 min (see Movie S1 in SI). The collagen microribbons were easily identified by the characteristic D-band structure of alternating overlap and gap regions.^21−23^ Similar features were observed in all of the maps, which enabled a complete characterization of the physical properties of the collagen structures during the growth process. At *t* = 0 s, about 10% of the mica surface is covered by collagen microribbons and microfibrils. At *t* = 512 s, about 80% of the mica is covered by microribbons ranging from 300 to 500 nm in width. A few microfibrils and thin microribbons are observed at an angle of 58° with respect to the dominant growth direction. This value is very close to the 60° angle between the main mica lattice directions. Thus, the dominant growth directions reflected an interplay between surface diffusion and electrostatic interactions between charged amino acid side chains and mica.^42^

Figure 1b shows the cross sections of the height, the elastic modulus, and the loss tangent along the lines marked at *t* = 510 s. The cross sections show an oscillating pattern that matches the 67 nm periodicity of the D-band (31 nm (overlap)/36 nm (gap)). The pattern observed in the height correlates with the changes observed in the elastic modulus and the loss tangent. The height varies from 4.4 nm (overlap) to 3.9 nm (gap). The elastic modulus and the loss tangent of the overlap and the gap regions are, respectively, 8.5 and 7.2 MPa and 0.60 and 0.82. Thus, overlap regions are thicker, stiffer, and dissipate less energy than gap regions. This tendency was similar to the one observed on thicker collagen samples.^43^ Bottom-effect corrections were applied^44^ to remove the influence of the rigidity support (∼50 GPa) on the elastic modulus measurements (see SI for details). The values of the elastic modulus reported here were similar to data obtained from AFM-based force–distance curves on thicker collagen fibrils (∼100 nm).^30,32,33^ Our raw data analyzed without the bottom-effect corrections gave elastic moduli values of about 50–100 MPa.

**Figure 2:**
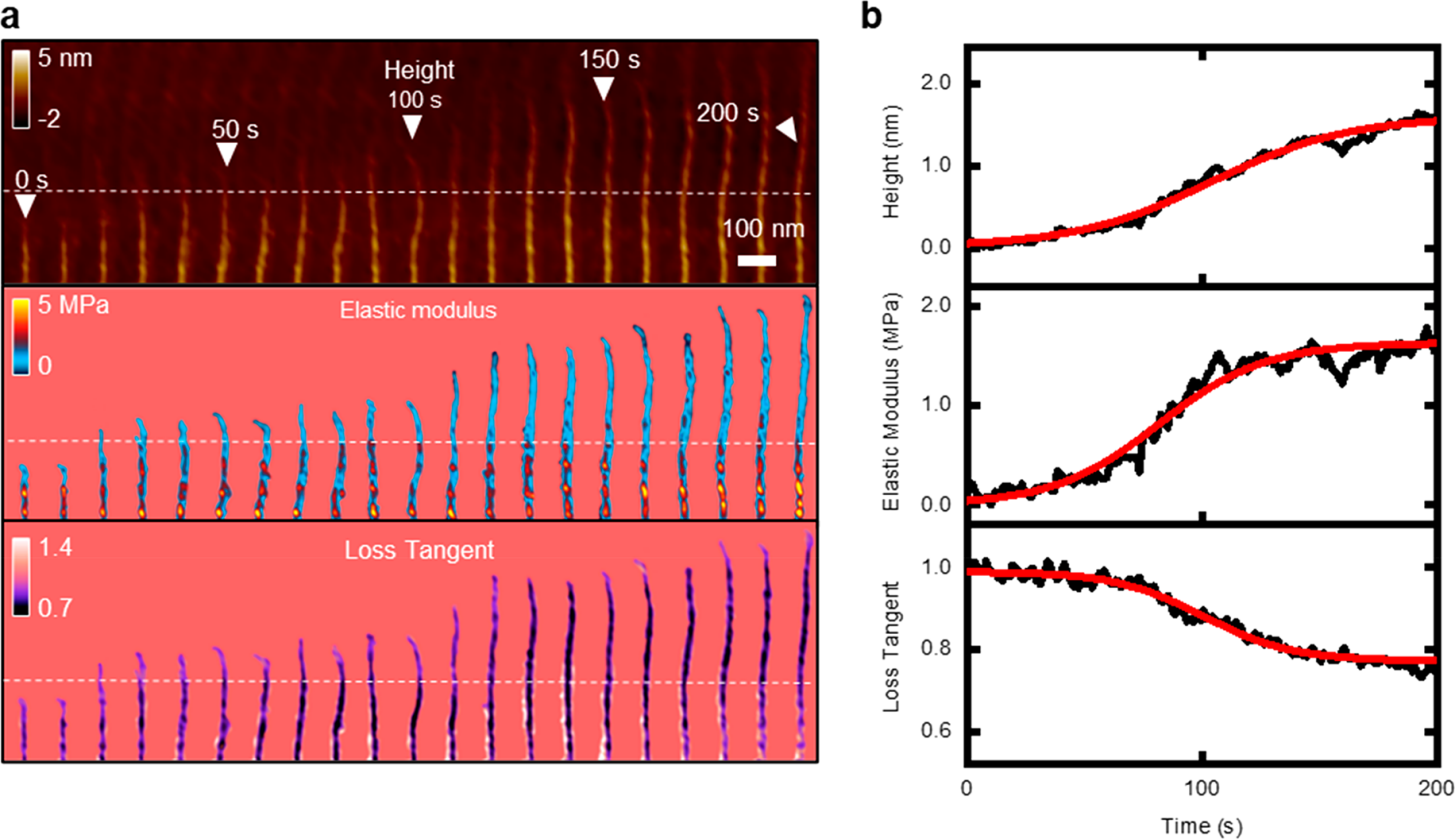
Growth dynamics of a single microfibril. (a) Kymograph of the height, the elastic modulus, and the loss tangent of a growing collagen microfibril. The images show the transition from the accretion of collagen precursors from the solution to the formation of a collagen microfibril (five tropocollagen molecules) with the D-band structure. (b) Cross sections along the dashed lines marked in the bimodal AFM images. At *t* = 0 s, there is no evidence of collagen precursors on the mica; at *t* = 150 s, the data showed the changes associated with the self-assembly of an emerging collagen structure (procollagen molecule); at *t* = 225 s, the collagen structure reached the diameter of a tropocollagen molecule, and the growth kinetics entered a stand still phase. The maps were obtained in buffer by applying a peak force of 1.2 nN on the collagen (18 nN on mica). Imaging rate, 1.12 fps (256 × 256 pixels, scanline of 300 Hz, see also Movie S2). Additional bimodal AFM data: *f*_1_ = 158 kHz, *k*_1_ = 0.35 nN/nm, *Q*_1_ = 1.5; *f*_2_ = 1159 kHz, *k*_2_ = 21 nN/nm, *Q*_2_ = 5.1; *A*_01_ = 7.1 nm, *A*_02_ = 0.2 nm, and *A*_1_ = 6.3 nm.

Figure 2a shows the evolution of the topography, the elastic modulus, and the loss tangent map of the tip of a single microfibril. The stage that preceded the formation of a tropocollagen molecule was characterized by monotonic changes in the mechanical properties and the topography. The nucleation and growth of collagen precursors produced an increase in height from 0 Å (bare mica) to 1.6 nm (Figure 2b). The latter value coincides with the expected diameter of a tropocollagen molecule.^22^ In the same process, the elastic modulus increased up to ∼1.6 MPa, while the loss tangent decreased from 1 (mica surface) to 0.79. To focus on the collagen growth, the Young’s values of the mica were removed from the analysis.

The HS bimodal AFM maps (Figures 1 and 2) enabled a complete characterization of the physical properties of collagen structures as a function of time. We identified four different stages of the collagen synthesis on mica. First, precursor collagen molecules from the buffer solution nucleated and grew. The most likely outcome from this process was the formation of a tropocollagen structure (a triple-helix molecule). This was followed by the packing of five tropocollagen molecules to form a microfibril. Collagen microfibrils grew and interacted among them to form 2D-packed collagen microribbons.

**Figure 3:**
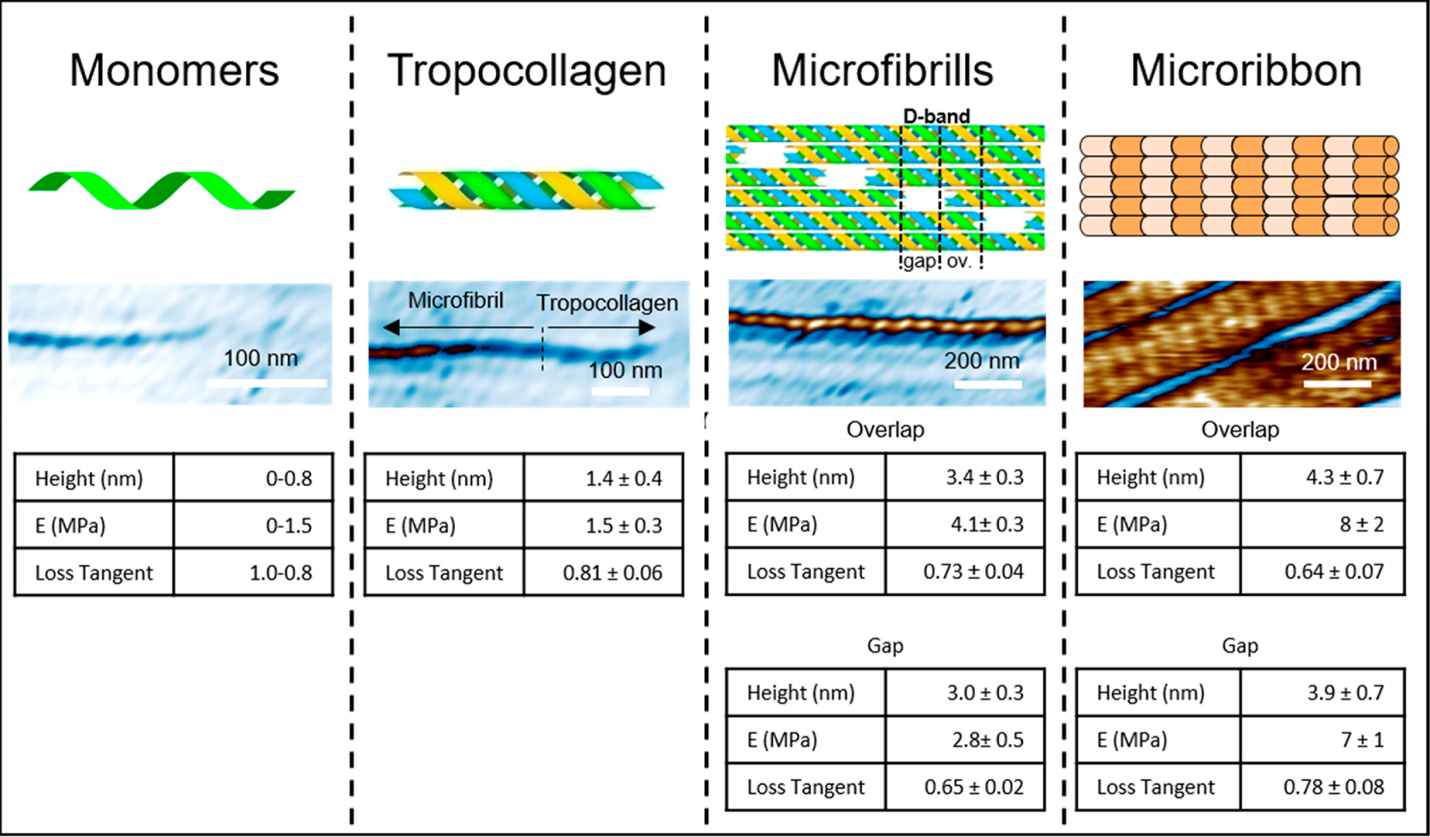
Physical properties in the first stages of the self-assembly of collagen. First stage: adsorption, nucleation, and growth of collagen precursors. Second stage: formation of tropocollagen. Third stage: assembly of tropocollagen molecules to form microfibrils. Fourth stage: merging and alignment of microfibrils to form microribbons. Those stages are characterized by different values in the physical properties.

Each of the above collagen structures had characteristic values in the physical properties (Figure 3). The process that led to the formation of a tropocollagen molecule from the nucleation of precursor molecules was characterized by monotonic changes in height, elastic modulus, and loss tangent. However, the growth dynamics from tropocollagen to microribbons was characterized by step-like changes. Thus, the height increased from 1.5 to 4.1 nm (mean value of gap and overlap regions); the elastic modulus changed from 1.5 to 7.5 MPa (mean), and the loss tangent decreased from 0.81 to 0.71 (mean). This small change was attributed to the lateral packing afforded by the microribbon structure. It was interesting to note that an increase in the complexity of the collagen structure was characterized by a reduction of its internal viscosity.

We measured the dependence of the elastic modulus values on the scanline frequency from 1 to 300 Hz. We did not observe a dependence on the imaging speed (SI Figure S4).

**Figure 4:**
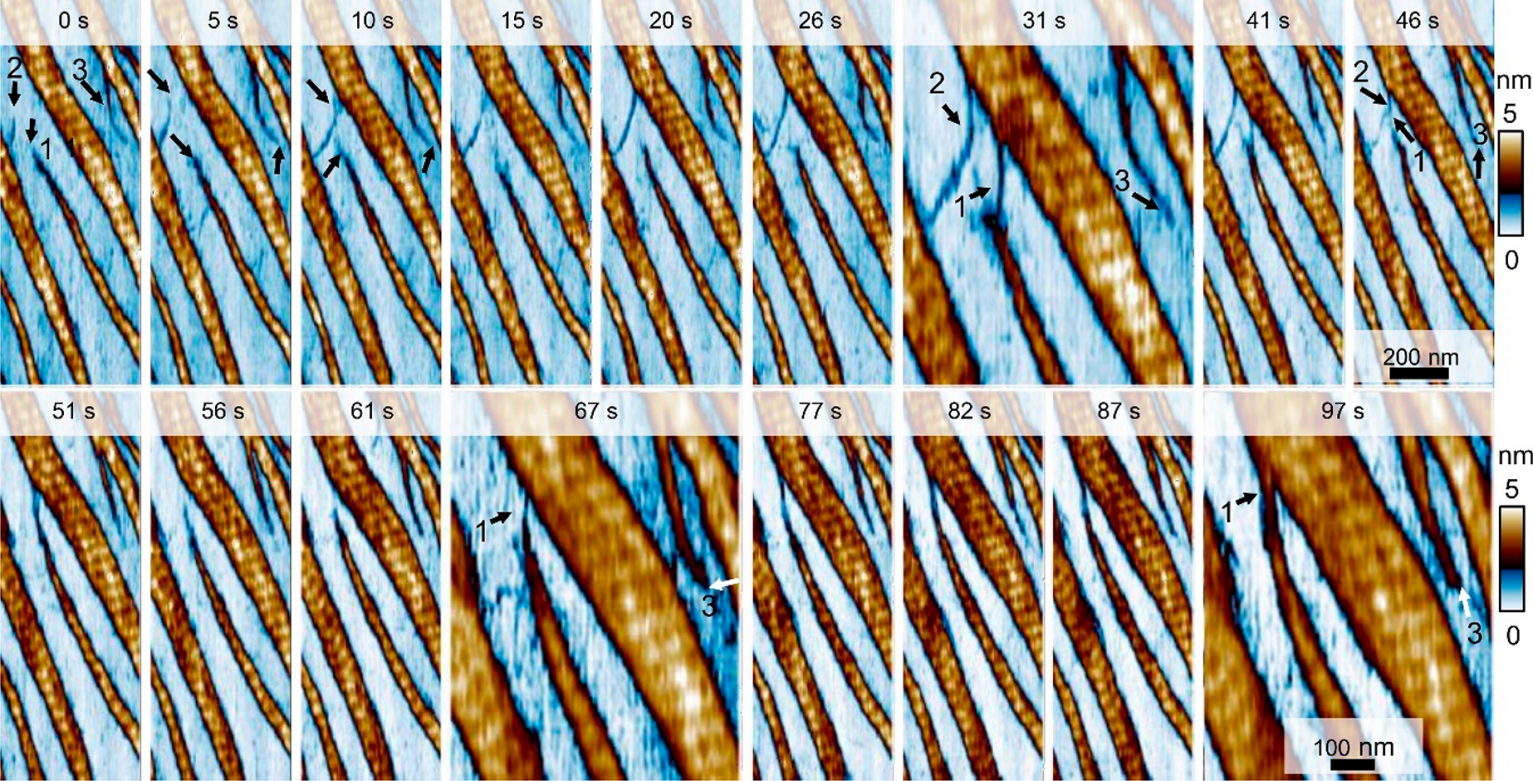
Pathways and growth dynamics of collagen self-assembly. Sequence of HS bimodal AFM images showing different collagen growth pathways marked as 1, 2, and 3 at *t* = 0 s. The tip (1) evolves to merge with a large microribbon at *t* = 97 s; (2) after a fast growth in length (*t* = 31 s), a disintegration process started that led to its disappearance (*t* = 51 s). The trajectory of (3) underlined the role of thermal fluctuations in the growth dynamics. Images were obtained in buffer by applying a peak force of 1 nN to the collagen microribbons (18 nN on mica). The images were extracted from images of larger area (1 μm × 1 μm). Imaging rate, 0.2 fps (512 × 512 pixels, scanline of 100 Hz). See Movie S3 in SI. Bimodal AFM data as in Figure 1.

The pathways and growth dynamics were monitored by acquiring images every 5 s (Figure 4 and Movie S3 at SI). At *t* = 0 s, we have marked three structures to follow their evolution: the tip of a microribbon (1), a spur coming out from a microribbon (2), and the tip of an emerging collagen structure (procollagen) (3). Initially, the tip (1) showed very little activity; from *t* = 26 to 31 s, it established contact with a tip coming from the microribbon above it. At *t* = 97 s, the tip (1) was fused with the microribbon.

The growth rate of the procollagen structure (2) was initially very fast; at *t* = 10 s, this structure bridged the gap between two microribbons. From *t* = 10 and 41 s, it entered into a stand still phase; at *t* = 46 s, there was evidence of some degradation which led to its disappearance (*t* = 51 s). Its components might have either returned to the solution or were recruited by the more active microfibril (1).

The structure labeled as (3) evolved from a microfibril (*t* = 20 s) to become a microribbon (*t* = 77 s). Its trajectory veered either to the right or left, revealing the influence of thermal fluctuations in the growth dynamics.

A recurrent question with nanomechanical mapping comes from the influence of the tip on the observed properties.^17^ To address this issue during collagen growth, we have also recorded the growth with HS-AFM operated in amplitude modulation. The topographic images obtained by HS-AFM were identical to the bimodal images (topography). We conclude that bimodal AFM operation did not introduce additional distortions on the measurement. In fact, the factor that might alter the growth dynamics is the force applied by the tip on the collagen, not the particular AFM operation mode used.

### Iso-time Maps

**Figure 5:**
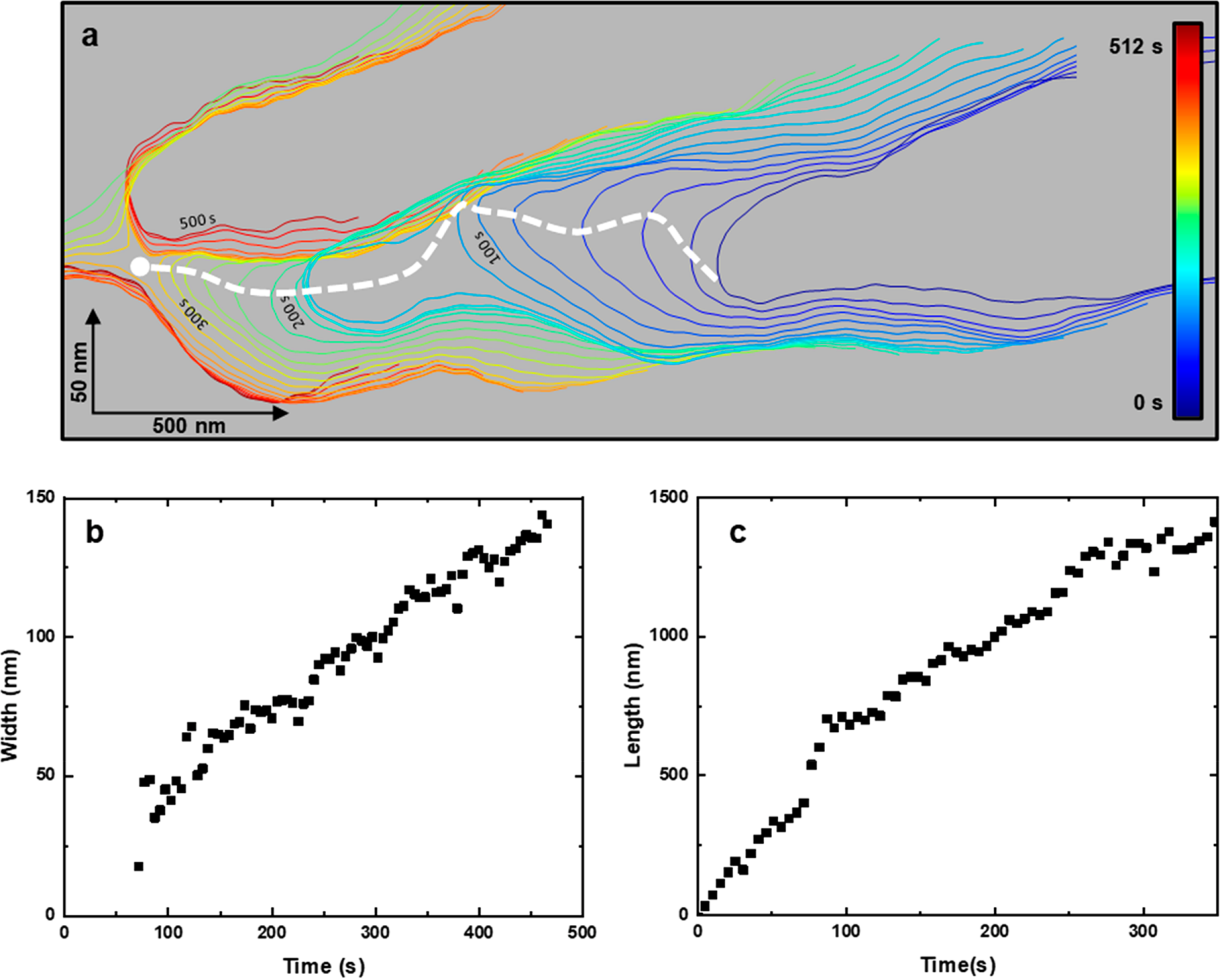
High-speed bimodal iso-time maps of collagen growth on mica. (a) Iso-time map shows the contour of a microribbon as a function of time. The trajectory of the growing collagen tip is marked by a dashed line. (b) Longitudinal and (c) lateral profiles as a function of time. The images were obtained in buffer by applying a peak force of 1.2 nN on the collagen (18 nN on mica). Imaging rate, 0.2 fps (512 × 512 pixels, scanline of 100 Hz, Figure S1 in SI). Bimodal AFM data as in Figure 1.

Details on the growth dynamics, in particular, the trajectory of the tip and the dominance of the axial growth comes from the bimodal iso-time maps (Figure 5). The bimodal iso-time map shows the evolution of the contour line of a microribbon and its tip’s trajectory as a function of time. The data to build an iso-time map comes from the bimodal AFM maps (see SI).

The trajectory of the microribbon’s tip was controlled by the accretion of collagen precursors, the interaction of the collagen with the mica lattice,^27,42^ and the thermal fluctuations. The tip veered to its right and merged with another microribbon. Upon contact, the tip moved to the left and the D-bands became aligned. The iso-time map cross sections (Figure 5b,c) showed the dominance of the axial growth. In this case, the ratio between the axial and lateral growth was about 10:1. However, this ratio depends on the concentration collagen precursors and the pH of the buffer solution.^24−27^ The saturation observed in the lateral growth comes from the merging of the two microribbons.

The analysis of the tip’s trajectory illustrates the role of the elastic modulus of the collagen microfibril during growth. The measured value of the elastic moduli of a microfibril was 3.5 MPa (mean value between gap and overlap regions). From it, we estimated the stiffness *k* of the tip of a collagen microfibril by
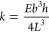
3which for a tip with an average width of *b* = 10 nm, thickness of *h* = 3.2 nm, and effective length of *L* = 100 nm gave ∼3 × 10^–6^ N/m. The amplitude of the fluctuations at ∼40 nm was determined by applying the equipartition theorem, *s* = (*k*_B_*T*/*k*)^1/2^, where *k*_B_ is Boltzmann’s constant and *T* is the temperature. Thermal fluctuations enabled a growing collagen structure to explore different regions on the mica surface, which facilitated the encounter of two or more microfibrils to form microribbons.

## Conclusions

We demonstrated that high-speed bimodal AFM provided quantitative mechanical property mapping in liquid at high speeds (5 fps), high-spatial resolution (sub-10 nm), and with a high elastic modulus sensitivity (0.1 MPa). The capabilities of the instrument were tested by analyzing the changes in the mechanical properties associated with the early stages of the collagen assembly on a mica surface. Those stages included the nucleation and the growth of precursor collagen molecules, the formation of tropocollagen, the assembly of collagen microfibrils, and their packing into collagen microribbons. The mechanical properties enabled the unambiguous identification of the early stages of collagen growth. The HS bimodal nanomechanical maps showed that some emerging collagen structures of a few hundreds of nanometers in length never matured into fully collagen microfibrils. The data showed evidence of the role of thermal fluctuations in driving the merging of microfibrils to form microribbons.

High-speed AFM enables direct imaging of biomolecule dynamics and processes at the subsecond time scale. By demonstrating the compatibility of high-speed imaging and nanomechanical mapping, the fields of nanoscience and microscopy are poised to experience a deep transformation. Morphological and mechanical property changes might be measured in real time and in native conditions.

## Materials and Methods

### HS Bimodal AFM

A home-built bimodal AM open-loop configuration and software were implemented for high-speed mapping in a commercial AFM platform (Cypher VRS, Oxford Instruments, USA). This configuration generated four observables, the amplitudes and phase shifts of the two excited modes (*A*_1_, *A*_2,_ ϕ_1,_ ϕ_2_). A feedback loop acts on the amplitude of the first eigenmode *A*_1_ to track the topography, whereas the observables of the second mode *A*_2_ and ϕ_2_ remain unlocked (SI). The application of the energy balance and the virial theorem for a Sneddon contact mechanics model enabled deduction of analytical expressions (see SI). Photothermal excitation was used to excite the vibration of the cantilever. The values of the free amplitudes depended on the specific cantilever; common values were *A*_01_ ∼ 10 nm and *A*_02_ ∼ 1 nm. The uncertainties in the observables (Δ*A*_1_, Δ*A*_2_, Δϕ_1_, Δϕ_2_) were, respectively, 120 pm, 15 pm, 2°, and 0.6°.

The experiments were performed with very small cantilevers (20 μm × 10 μm × 190 nm) (USC-F0.3-k0.3, NanoAndMore, Germany). Typical values of the resonant frequencies, force constants, and quality factors in liquid were, respectively, *f*_1_ ∼ 120 kHz, *f*_2_ ∼ 1 MHz; *k*_1_ ∼ 0.3 N/m, *k*_2_ ∼ 15 N/m; and *Q*_1_ ∼ 1.5 and *Q*_2_ ∼ 5.1. The exact values of the parameters for each image, and the calibration protocols are reported in the SI. Force constants were calibrated using the method described in ref (45).

The HS bimodal AFM has a *z*-feedback bandwidth of 100 kHz, an optical detector bandwidth of 7 MHz, and a lock-in sampling rate of 500 kHz. A typical mechanical bandwidth (∼*QT*/π) in the experiments (data of Figure 2) was about 324 kHz (∼3 μs), whereas the pixel time was 6.5 μs.

Using a cantilever with a *f*_1_ ≈ 647 kHz and *Q*_1_ = 1.5 (see SI), we generated a nanomechanical map of collagen nanoribbons at an imaging rate of 5 fps (Movie S4, 368 × 96 pixels, scanline of 543 Hz). In that case, the pixel time was 2.1 μs while the tip’s period was 1.5 μs. However, most of the bimodal AFM data were acquired at an imaging rate of 1.2 fps (256 × 256 pixels, scanline of 300 Hz). The size of the collagen structures and the growth kinetics did not require the use of higher rates.

### Collagen and Buffer

The imaging buffer consisted of phosphate-buffered saline (Sigma-Aldrich) with 300 mM KCl (Sigma-Aldrich), pH 7.4. Monomeric bovine collagen type I (PureCol, CellSystems GmbH) was used. The as-received collagen solution was diluted to a concentration of 3.0 μg mL^–1^ using the imaging buffer and rapidly injected into a freshly cleaved muscovite mica disk (Grade V-1, Alpha Biotech Ltd.) placed inside a fluid chamber. High-speed bimodal imaging started without further delays. The measurements were performed at a temperature of 22 °C.

### Image Processing and Data Analysis

Data and videos were processed using a home-built software written in python. The height, the elastic modulus, and the loss tangent channels were corrected for thermal drift. Trace and retrace signals were averaged. The frames were combined with ffmpeg to produce the videos.

## Supporting Information Available

Supporting Figures S1–S5: HS-bimodal AFM topography data to generate the iso-time maps, tip’s radius determination, bottom-effect corrections, elastic modulus at different imaging speeds, bimodal AFM images of collagen fibrils acquired at 5 fps; experimental methodology details and derivation of the bimodal AFM equations (PDF)
Movies S1: High-speed bimodal AFM maps of the early stages of the growth collagen nanoribbons on mica (AVI)
Movie S2: Growth dynamics of a single microfibril (AVI)
Movie S3: Pathways and growth dynamics of collagen self-assembly (AVI)
Movie S4: Bimodal AFM imaging of collagen fibrils taken at 5 fps (AVI)

## Supplementary Material

nn0c10159_si_001.pdf

nn0c10159_si_002.avi

nn0c10159_si_003.avi

nn0c10159_si_004.avi

nn0c10159_si_005.avi
