# Supplementary material for: High-Speed Nanomechanical Mapping of the Early Stages of Collagen Growth by Bimodal Force Microscopy
Source: ACS Nano. 2021 Jan 7;15(1):1850–7. doi: 10.1021/acsnano.0c10159 (PMC8477367; doi:10.1021/acsnano.0c10159)
Supplement: Supplementary file 1 — nn0c10159_si_001.pdf [file nn0c10159_si_001.pdf]

Supporting Information

High-Speed Nanomechanical Mapping of the Early-Stages of Collagen Growth by Bimodal Force Microscopy

Victor G. Gisbert†, Simone Benaglia†, Manuel R. Uhlig†, Roger Proksch‡, Ricardo Garcia†*

†Instituto de Ciencia de Materiales de Madrid, CSIC, c/ Sor Juana Ines de la Cruz 3, 28049 Madrid, Spain

‡Asylum Research an Oxford Instruments Company, Santa Barbara, California 93117, United States

* Email: r.garcia@csic.es.

List of contents

- **Movie captions (Movie 1-4)**
- **Figure S1**
- **Figure S2**
- **Figure S3**
- **Figure S4**
- **Figure S5**
- **Elastic modulus and loss tangent values from bimodal AFM data (AM-Open Loop)**
- **Tip's radius determination**
- **Bottom-effect correction for the determination of nanomechanical properties**
- **Cantilever calibration**
- **Supplementary References**

Movie Captions

Movie S1. High-speed bimodal AFM maps of the early-stages of the growth collagen nanoribbons on mica. The panels show the height signal, elastic modulus and loss tangent. The images showed in Fig. 1 were extracted from the movie. The images were obtained at 100 Hz, 3μm scan size, 512x512 pixels and 0.2 fps. The cantilever parameters (USC-F0.3-k0.3, NanoAndMore). were f_1 = 132 kHz, k_1 = 0.18 nN/nm, Q_1 = 1.7 for the first mode and f_2 = 1045 kHz, k_2 = 12 nN/nm, Q_2 = 5.3 for the second mode. The measurement was performed with a free amplitude A_{01} = 10 nm, A_{02} = 1.5 nm and A_1 = 9 nm. The maximum force F_{peak} exerted on the collagen was of 1 nN.

Movie S2. Growth dynamics of a single microfibril. The images showed in Fig. 2 were extracted from the movie. The imaging frequency (fast direction) was 300 Hz, 3 μm scan size, 256 x 256 pixels and 1.2 fps. The cantilever parameters (USC-F0.3-k0.3, NanoAndMore). were f_1 = 158 kHz, k_1 = 0.35nN/nm, Q_1 = 1.5 for the first mode and f_2 = 1159 kHz, k_2 = 21 nN/nm, Q_2 = 5.1 for the second mode. The measurement was performed with a free amplitude A_{01} = 7.1 nm, A_{02} = 0.2 nm and A_1 = 6.3 nm. The maximum force F_{peak} exerted on the collagen was of 1 nN.

Movie S3. Pathways and growth dynamics of collagen self-assembly. The images showed in Fig. 4 were extracted from this movie. The images were obtained at 100 Hz, 3μm scan size, 512x512 pixels and 0.2 fps. The cantilever parameters (USC-F0.3-k0.3, NanoAndMore). were f_1 = 132 kHz, k_1 = 0.18 nN/nm, Q_1 = 1.7 for the first mode and f_2 = 1045 kHz, k_2 = 12 nN/nm, Q_2 = 5.3 for the second mode. The measurement was performed with a free amplitude A_{01} = 10 nm, A_{02} = 1.5 nm and A_1 = 9 nm. The maximum force F_{peak} exerted on the collagen was of 1 nN.

Movie S4: HS-Bimodal AFM Imaging of collagen fibrils taken at 5 fps. The images were obtained at 543 Hz, 1 μm scan size and 96x368 pixels. The cantilever parameters (USC-F1.2-k0.15) were f_1 = 647 kHz, k_1 = 0.08 N m^{-1}, Q_1 = 1.5 for the first mode and f_2 = 5.6 MHz, k_2 = 8 N m^{-1} for the second mode. The measurement was performed with a free amplitude A_{01} = 11.0 nm, A_1 = 8.5 nm, A_2 = 1.0 nm. The maximum force F_{peak} exerted on the collagen was of 1 nN.

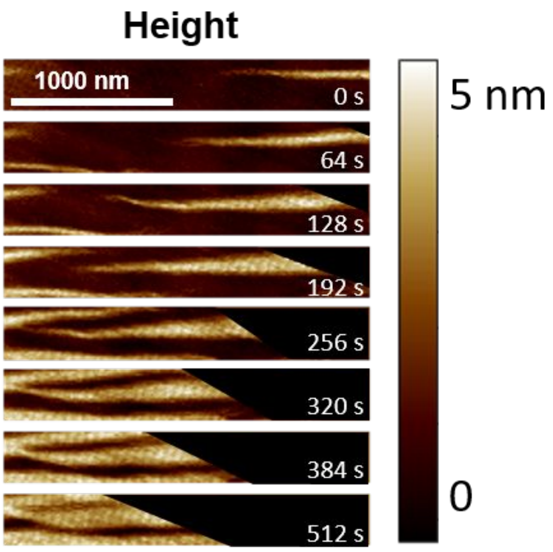

Supplementary Figure 1. HS-bimodal AFM topography data to generate the iso-time maps The images were obtained at 100 Hz, 3 μm scan size, 512 x 512 pixels and 0.2 fps. The san time was of 512 seconds. The cantilever used was a USC-F0.3-k0.3. The cantilever parameters were f_1 = 132 kHz, k_1 = 0.18 nN/nm, Q_1 = 2.4 for the first mode and f_2 = 1045 kHz, k_2 = 12 nN/nm, Q_2 = 5.7 for the second mode. The measurement was performed with a free amplitude A_{01} = 10 nm, A_{02} = 1.5 nm and A_1 = 9 nm. The maximum force F_{peak} exerted on the collagen was of 1 nN.

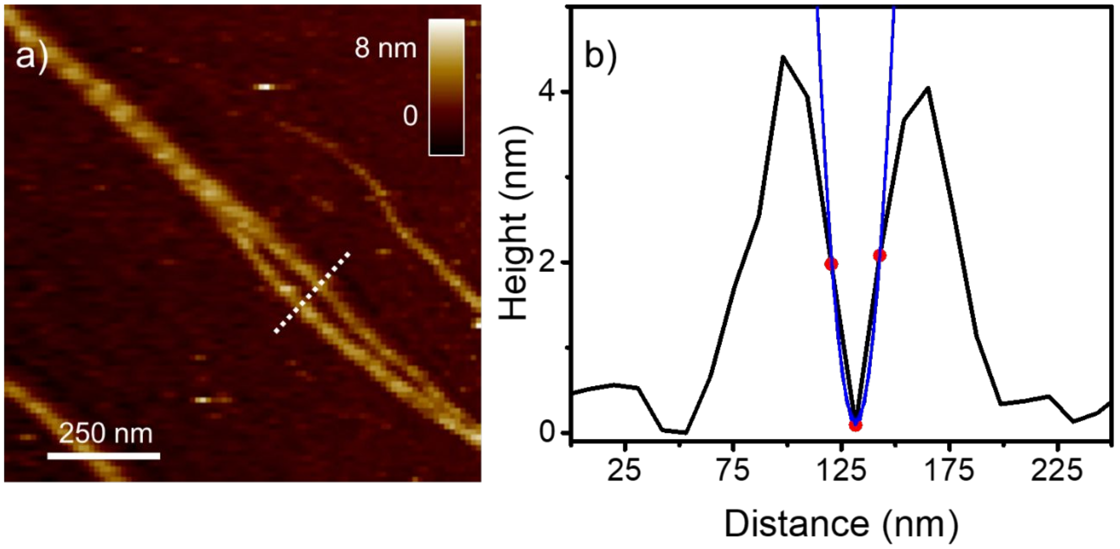

Supplementary Figure 2. Tip's radius determination. (a) Frame of a bimodal high-speed height sequence. A microribbon split in half is defined as the smallest feature where mica and collagen are clearly recognizable. (b) The black line is the cross section referred to the dot line in (a). The blue line refers to the fitting obtained through the three points marked in red.

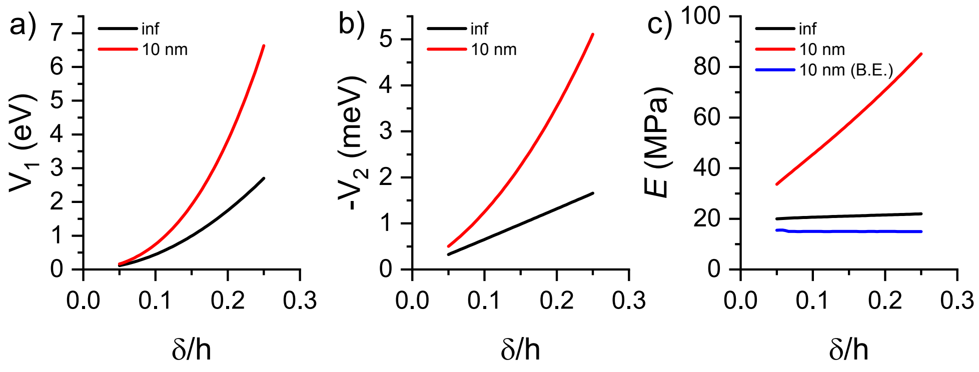

Supplementary Figure 3: Bottom-effect corrections. **a.** Dependence of the virial of mode 1 as a function of the ratio between the height and indentation for a semi-infinite sample and for a 10 nm thick sample (E = 10 MPa in both cases). **b.** Dependence of the virial of mode 2 as a function of the indentation ratio for a semi-infinite sample and for a 10 nm thick sample. **c.** Young's modulus for three samples: semi-infinite, 10 nm thick (no corrections) and a finite sample with bottom-effect corrections. The data comes from bimodal AFM measurements.

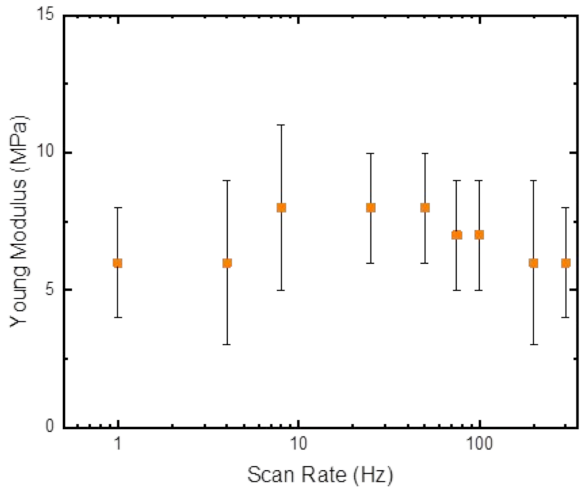

Supplementary Figure 4: Elastic modulus at different imaging speeds. **a.** Elastic modulus maps of collagen were obtained at different scanline rates from 1 to 300 Hz. The measurements were performed on the same collagen sample. The images were obtained at 1μm scan size and 512x512 pixels. The cantilever parameters (USC-F0.3-k0.3, NanoAndMore). were f_1 = 137 kHz, k_1 = 0.33 nN/nm, Q_1 = 2.1 for the first mode and f_2 = 1083 kHz, k_2 = 20 nN/nm, Q_2 = 5.6 for the second mode. The measurement was performed with a free amplitude A_{01} = 5.7 nm, A_{02} = 0.3 nm and A_1 = 4.4 nm. The maximum force F_{peak} exerted on the collagen was of 2 nN.

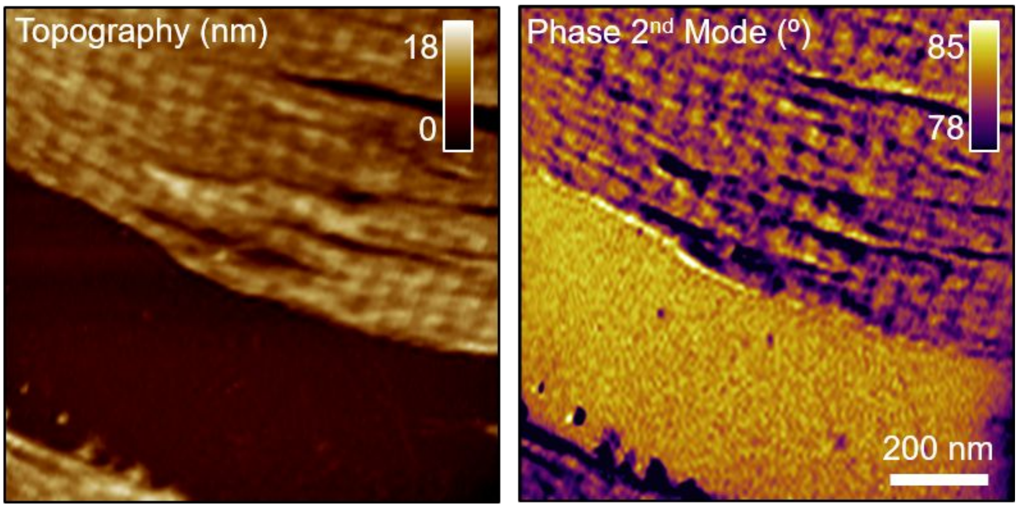

Supplementary Figure 5: HS-Bimodal AFM Imaging of collagen fibrils acquired at 5 fps (96x368 pixels at a scanline rate of 543 Hz, see video S4 in SI). The bimodal AFM parameters were A_{01} = 11.0 nm, A_1 = 8.5 nm, A_2 = 1.0 nm and F_{max} = 1 nN. Images were obtained with a USC-F1.2-k0.15 cantilever (f_{01} = 647 kHz, k_1 = 0.08 N m^{-1}, Q_1 = 1.5, f_{02} = 5.6 MHz, k_2 = 8 N m^{-1}). Experiment performed in liquid.

Elastic modulus and loss tangent values from bimodal AFM data (AM-open loop)

The expression to determine the compressive elastic modulus and the loss tangent from bimodal AFM were deduced by applying energy balance and virial theorem considerations to the tip's motion. The general process was developed previously for a semi-spherical tip of radius *R*.[1-5]

The elastic modulus was determined by

$$E_{\text{eff}} = \sqrt{\frac{8}{R\,A_1}}\,\frac{2Q_1A_1}{k_1A_{01}\cos\phi_1}\left(\frac{k_2A_{02}\cos\phi_2}{2Q_2A_2}\right)^2 \quad \text{(s1)}$$

$$\frac{1}{E_{\text{eff}}} = \frac{1-\nu_{\text{coll}}^2}{E_{\text{coll}}} + \frac{1-\nu_{\text{tip}}^2}{E_{\text{tip}}} \approx \frac{1-\nu_{\text{coll}}^2}{E_{\text{coll}}} = \frac{0.938}{E_{\text{coll}}} \quad \text{(s2)}$$

where E_{tip} = 150 GPa and ν(collagen)=0.25.

We assumed that the energy dissipation process is detected by the first mode. The loss tangent represents the ratio between the energy dissipated in sample and the energy store in the cantilever-tip system in one cycle.[6] It was calculated by

$$\tan\rho = \frac{1}{2\pi}\frac{E_{\text{dis}}(\text{mode }1)}{V(\text{mode }1)} = \frac{\sin\phi_1 - {A_1}/{A_{01}}}{\cos\phi_1} \quad \text{(s3)}$$

where E_{dis} is the energy dissipated and V_1 is the virial of mode 1.

$$E_{\text{dis}} = \frac{\pi k A_{01}}{Q_1}(A_{01}\sin\phi_1 - A_1) \quad \text{(s4)}$$

$$V_1 = -\frac{k_1 A_1 A_{01}}{2Q_1}\cos\phi_1 \quad \text{(s5)}$$

The deformation (maximum value) produced by the tip's force was determined by

$$\delta = \frac{\frac{k_1 A_{01}\cos\phi_1}{Q_1} \quad \frac{Q_2 A_2}{k_2 A_{02}\cos\phi_2}}{} \quad \text{(s6)}$$

We note that Eq. s3 and s4 provide accurate determination of the loss tangent and the energy dissipated for high quality factor cantilevers. In liquid, the first mode might lose energy through different processes, interaction with the sample, the liquid and energy losses to higher harmonics.[7] The latter processes were not been taking into account in the above expressions. This effect is particular relevant when comparing loss tangent values between mica (~50 GPa) and collagen (~5 MPa). The tip-sample interaction force on mica will generate higher harmonics components. The amplitudes of those harmonics will be significantly larger than the amplitudes of the harmonics generated on collagen. However, Eq, s3 provides a good approximation to estimate the dissipative properties of the collagen structures because the change in Young's modulus is of a few MPa. Those differences do not generate significant changes in the high harmonic components.

Tip's radius determination

The elastic modulus depends on the tip's radius as defined in equation s1. We have applied an *in situ* process to determine the tip's radius based on the spatial resolution given by the bimodal AFM image. Fig. S2a shows a HS-bimodal AFM image (topography) of a microribbon split in two. The tip's radius is determined by finding the best fit to the cross-section (Fig. S2b). The points selected to perform the fitting are marked in red in Fig. S2b (the minimum and the tangent points). A radius R=33 nm was obtained for Fig. S2a.

Bottom-effect correction for the determination of nanomechanical properties

The force exerted by an AFM on a soft and very thin material such as collagen microfibrils bears the influence of the elastic properties of the solid support.[8] To account for this effect in the determination of the elastic modulus of the collagen we have used the following expression for the force exerted by the tip,[7]

$$F_{\text{ts}}(\delta, E_{\text{eff}}, \text{h}) = \frac{16}{9}E\sqrt{R}\delta^{3/2}\left[1 + \frac{1.133\sqrt{\delta R}}{h} + \frac{1.497\delta R}{h^2} + \frac{1.469\,\delta\,R\sqrt{\delta R}}{h^3} + \frac{0.755\,\delta^2\,R^2}{h^4}\right] \quad \text{(s7)}$$

δ is the deformation (indentation) and h the thickness of the undeformed collagen. Equation s7 was introduced in the virials[4] of modes 1 and 2 to determine the elastic modulus and the indentation.

Cantilever calibration

The cantilever parameters were calibrated after the experiment. An extensive explanation of the fitting procedure has already been described.[3] The inverse optical lever sensitivity (nm V^{-1}) of the first mode was calibrated by acquiring a deflection–distance curve on a stiff surface (muscovite mica). With a measurement of the power spectral density (PSD) the spring constant of the first mode was calibrated, fitting it with the single harmonic oscillator (SHO) model. Thus, the resonance frequency of the second mode, f_2, is measured from the PSD and the corresponding force constant is calculated with the stiffness–frequency power law relationship given by,[9]

$$k_2 = k_1\left(\frac{f_2}{f_1}\right)^{\zeta} \quad \text{(s9)}$$

assuming $\zeta = 2$. Once k_2 is known, the PSD is fitted with the SHO where the peak of the second resonance frequency is to obtain the corresponding sensitivity and Q_2.

Supplementary References

1. Herruzo, E. T.; Garcia, R. Theoretical Study of the Frequency Shift in Bimodal FM-AFM by Fractional Calculus. *Beilstein J. Nanotechnol.* **2012**, *3*, 198–206.
2. Labuda, A.; Kocuń, M.; Meinhold, W.; Walters, D.; Proksch, R. Generalized Hertz Model for Bimodal Nanomechanical Mapping. *Beilstein J. Nanotechnol.* **2016**, *7*, 970–982.
3. Benaglia, S.; Gisbert, V. G.; Perrino, A. P.; Amo, C. A.; Garcia, R. Fast and High-Resolution Mapping of Elastic Properties of Biomolecules and Polymers with Bimodal AFM. *Nat. Protoc.* **2018**, *13*, 2890–2907.
4. Lozano J. R.; Garcia, R. Theory of Multifrequency in Bimodal Atomic Force Microscopy *Phys. Rev. Lett.* **2008**, *100*, 076102.
5. Gómez, C. J.; Garcia, R. Determination and Simulation of Nanoscale Energy Dissipation Processes in Amplitude Modulation AFM. *Ultramicroscopy*, **2010**, *110*, 626-633.
6. Proksch, R.; Kocun, M.; Hurley, D.; Viani, M.; Labuda, A.; Meinhold, W.; Bemis, J. Practical Loss Tangent Imaging with Amplitude-Modulated Atomic Force Microscopy. *J. Appl. Phys.* **2016**, *119*, 134901.
7. Proksch, R.; Yablon, D. G. Loss Tangent Imaging: Theory and Simulations of Repulsive-Mode Tapping Atomic Force Microscopy. *Appl. Phys. Lett.* **2012**, *100*, 073106.
8. Garcia, P. D.; Garcia, R. Determination of the Elastic Moduli of a Single Cell Cultured on a Rigid Support by Force Microscopy. *Biophys. J.* **2018**, *114*, 2923–2932.

9. Labuda, A.; Kocun, M.; Lysy, M.; Walsh, T.; Meinhold, J.; Proksch, T.; Meinhold, W.; Anderson, C.; Proksch, R. Calibration of Higher Eigenmodes of Cantilevers. *Rev. Sci. Instrum.* **2016**, *87*, 073705.
